# The Effectiveness of Minimally Invasive Techniques in the Treatment of Patellar Tendinopathy: A Systematic Review and Meta-Analysis of Randomized Controlled Trials

**DOI:** 10.1155/2020/8706283

**Published:** 2020-09-05

**Authors:** M. P. López-Royo, M. Ortiz-Lucas, E. M. Gómez-Trullén, P. Herrero

**Affiliations:** ^1^iPhysio Reaserch Group, Universidad San Jorge, Campus Universitario, Autov. A23, Km 299, Villanueva de Gállego, Zaragoza 50830, Spain; ^2^Universidad de Zaragoza, Facultad de Ciencias de la Salud, Dpto. de Fisiatría y Enfermería, C/Domingo Miral s/n, Zaragoza 50009, Spain

## Abstract

The aim was to determine the effectiveness of minimally invasive techniques (MIT) in patients with patellar tendinopathy. Database searches were performed for randomized controlled trials (RCTs) in electronic databases (WOS, Cochrane Central, SportDiscus, and Medline via PubMed and PEDro). The inclusion criteria used were published in English or Spanish and involving adults with patellar tendinopathy (pain on the inferior pole of the patella for a minimum of 3 months), with at least one group receiving MIT. The quality of the relevant RCTs was evaluated using the PEDro scale. The primary outcome was functionality using the VISA-p questionnaire. Secondary outcome was focused on pain. A total of 1164 studies were screened for possible inclusion in our systematic review. Finally, 10 RCTs were included with a total of 326 individuals. Five RCTs were included in the meta-analysis. The quality assessment revealed that all the studies included were considered to possess high methodological quality. All studies analyzing MIT such as platelet-rich plasma (PRP), dry needling, or skin-derived tenocyte-like cells, when combined with exercise, proved to be effective for patellar tendinopathy. Moreover, the PRP technique with doses greater than 4 mL together and combined with an exercise program lasting over 6 weeks obtained better results in functionality and pain than other treatments in the short term. However, in the long term, dry needling and skin-derived tenocyte-like cells are more effective than PRP. Although the infiltration of drugs was effective at posttreatment, these improvements were not maintained over time and may have secondary effects. Although there are no RCTs analyzing the effectiveness of MIT like percutaneous needle electrolysis, there has been an increasing number of publications achieving excellent results in the last years. However, it is necessary to develop RCTs analyzing not only the effect but also comparing the effectiveness between different MIT such as dry needling and percutaneous needle electrolysis.

## 1. Introduction

Patellar tendinopathy (PT) is a degenerative disease of the patellar tendon, in which the patient complains of pain in the inferior pole of the patella. Also, a disturbed collagen distribution, changes in vascularity and cellularity, increased thickness of tendon, and incompletely healed tendon microruptures are common changes observed in patients with this pathology [1–3]. The major cause of this injury is overuse during activities that involve jumping, running, and rapid changes of direction, which are very common movements in sports such a basketball and volleyball. Specifically, in nonprofessional athletes, the prevalence varies between 2.5% in soccer players and 14.4% in volleyball players. In contrast, in both elite sports, 40–50% of professional athletes are affected [4–6]. The diagnosis of PT is typically based on medical history and clinical findings [7]. Imaging techniques such as Color Doppler sonography (CD) and magnetic resonance imaging (MRI) are valuable tools to confirm the diagnosis and provide guidance for treatment [8].

Conventional treatments such as eccentric exercise (EE) programs for the quadriceps tendon, as a part of standard treatment, has demonstrated to have positive results, to the point that many authors have defended this technique as the gold standard for the treatment of tendinopathies [9–12]. A recent systematic review reported that the best exercise for improving knee function and reducing pain in the long term is based on EE and heavy slow resistance exercises (HSR) [13]. In addition, recent research has focused on minimally invasive techniques (MIT) using needles, with high expectations of success. These techniques seek a rapid regeneration of the injured tendon and the relief of chronic pain [4, 8, 14–16]. Their effect is based on stimulating the cellular activity and the production of collagen, as well as influencing the biomechanics of tendons to obtain restructuration of the matrix.

It can be classified according to whether they inject any substances or not. Regarding techniques that do not infiltrate any substances, good clinical results are being reported with dry needling (DN) [17] and percutaneous needle electrolysis (PNE) [4, 18–20], which adds the effect of the galvanic current to the mechanical effect of the needle. PNE combined with EE programs offer excellent results in terms of the clinical and functional improvement of the patellar tendon in the short-term and a rapid return to the previous level of activity [4, 18]. Concerning techniques based on the infiltration of substances, it is possible to differentiate between pharmacological or nonpharmacological agents. The most used technique is platelet-rich plasma (PRP) [21–23]. Literature reviews carried out based on the application of the PRP technique on other tendons have concluded that there are no significant differences between PRP and DN or placebo at the 6-months follow-up, although it seems that there may be small differences depending on the injured tendon [24]. Moreover, some reviews and meta-analysis have analyzed the effect of PRP in PT versus other techniques, regardless of whether they are conservative or invasive treatments, finding that PRP seems to be a more effective treatment in the long term than other treatments, such as extracorporeal shockwave therapy (ESWT) [15, 25, 26]. However, when compared to other invasive treatments such as cell therapy or DN, the results for PRP are worse regarding functionality in the long term, whereas these other techniques have a promising future for this pathology [27, 28]. Most studies conclude that more studies, specifically for randomized controlled trials (RCTs), are necessary to make accurate conclusions with scientific rigor, as most of the included studies reviewed are of low scientific quality.

A variety of MIT are being used in clinics for the treatment of PT, either used alone or as a complement to other conservative treatments. However, evidence-based therapies are limited, and there is no consensus regarding which of these is the most effective. The variability of the types of studies, together with the low quality of the same, lead us to consider the need for a review and meta-analysis of high-quality RCT on MIT for the treatment of PT.

Thus, our primary aim was to systematically review the effectiveness of MIT for functionality and pain of the PT in humans. Where possible, meta-analysis of outcome data on pain and function was performed.

## 2. Materials and Methods

A systematic review and meta-analysis were performed according to the Preferred Reporting Items for Systematic Review and Meta-Analysis (PRISMA) [29]. The review was registered in the international PROSPERO database (CRD42015025801). The software used to assemble all the papers included in this review is EndNote X7 v17.0.1.

### 2.1. Data Sources and Searches

We conducted a systematic literature search in Web of Science, Cochrane Central, SportDiscus, and Medline via PubMed and PEDro. The database search was conducted by two reviewers (MPLR and EMGT) in January 2018 with the final search conducted in September 2019, using the following search terms: patellar ten∗, patellar ligament, jumper's knee, chronic patellar ten∗, dry need∗, intratissue percutaneous electrolysis, acupuncture, electroacupuncture, mesotherapy, injection, injectable∗, puncture, and infiltrate∗. Search strategy for Medline is described in detail in Appendix 1.

Two independent reviewers (MPLR and EMGT) screened the titles and/or abstracts and full texts according to the stated inclusion criteria. Discrepancies were discussed at a consensus meeting, and the opinion of a third independent reviewer (MOL) was sought if agreement could not be achieved. Reviewers were not blinded to information regarding the authors, journal of origin, or outcomes for each paper reviewed.

### 2.2. Study Selection

The inclusion criteria were as follows: (a) RCT; (b) English or Spanish; (c) involving adults; (d) with a diagnosis of chronic patellar disease (minimum of 3 months of pain in the inferior pole of the patella); and (e) at least one group receiving MIT. We excluded articles in which the sample was under 18 years old, non-RCTs (quasiexperimental trials, case series, and observational studies), and those in which subjects had received a previous surgical intervention.

In studies with more treatment groups in which patients underwent different procedures, each treatment group was analyzed independently according to the treatment applied and always compared with the group receiving MIT.

### 2.3. Data Extraction

The primary outcome evaluated was functionality using the Victorian Institute of Sport Assessment of Patellar Questionnaire (VISA-p) [30]. Secondary outcome measures focused on pain.

A standardized electronic data extraction form was developed to obtain key information relevant to this review. Data extraction for each article was performed by two authors (MPLR and EMGT), with a third reviewer (MOL) solving potential discrepancies, and including the following: (1) sample characteristics (sample age and sample sex); (2) methodological characteristics (study design, number of groups, associated interventions, time of measurements, and assessment tools); (3) main results; and (4) conclusions.

Finally, a meta-analysis was performed grouping the RCTs according to the technique used. Means, standard deviation (SD), and sample sizes for pain and function were extracted for each group at short-term and medium/long-term follow-up. When data were not extractable or expressed in other form instead of mean and SD, a total of three attempts were made contacting with the corresponding author to request the information by e-mail, and in case of not getting an answer of the study data, were not used for the meta-analysis. To analyze the benefit of this treatment, studies without pre- and postintervention data were excluded.

### 2.4. Risk of Bias Assessment

The quality of the relevant RCTs was evaluated using the PEDro scale. Eleven PEDro criteria were used to evaluate the quality of the retrieved studies. Each satisfied item (except for item 1, which, unlike other scale items, pertains to external validity) contributes one point to the total PEDro score (range 0–10 points). Each criterion was assessed with the alternatives ‘‘Yes” (1 point) or ‘‘No” (0 points). Items 2–9 assess internal validity, and the last two items (10 and 11) reveal whether the statistical information presented in the study is enough to perform a correct interpretation of the outcomes. Articles fulfilling at least 6/10 positive criteria were considered to be of “good quality,” studies with 4-5/10 positive criteria were considered to be “average quality,” and articles with less than 4 points were considered “poor quality” [31]. Two reviewers (MPLR and EMGT) separately evaluated the quality of the studies using the PEDro scale, and when they could not reach agreement, a third independent reviewer was consulted.

### 2.5. Statistical Analysis

The random effects model was used for all meta-analyses. The VISA-p score and visual analog scale (VAS) mean differences and SD for each group (between pre and posttreatment and between pretreatment and the follow up) were collected for this purpose. Heterogeneity between studies was assessed using the Cochran's Q test, and the I^2^ index was used to quantify the amount of heterogeneity, with a value greater than 50% indicating substantial heterogeneity [32, 33]. Additional intragroup meta-analyses of each treatment differences of means were performed to explain the heterogeneity found. Also, subgroup analyses were performed according to dose (≤ or >4 mL), amount of exercise (≤ or >6 weeks), and basal VISA-p score (< or ≥48). Prior to this, scatter plots of possible factors of heterogeneity were drawn to detect suitable variables for subgroup analyses and their cut off values.

## 3. Results

### 3.1. Characteristics of Included Trials

**Figure 1:**
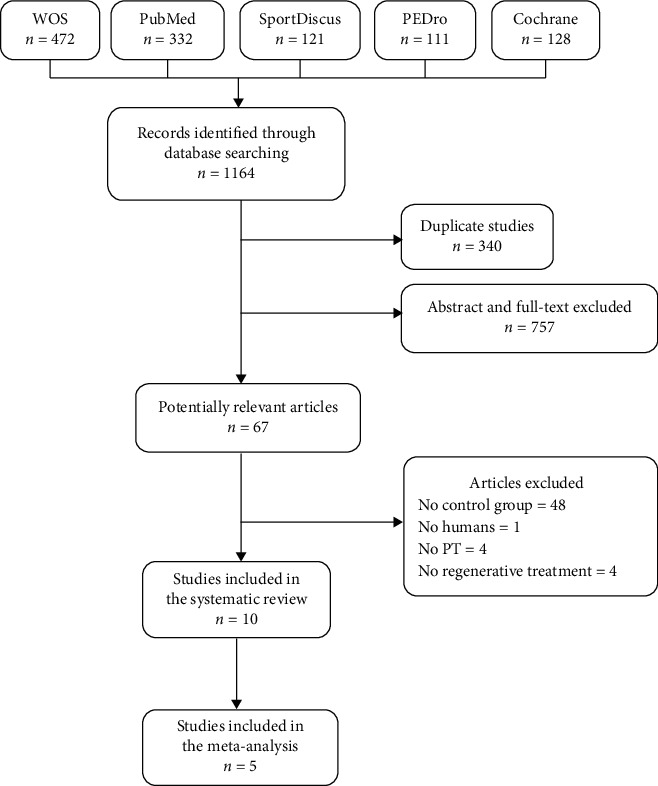
PRISMA flow diagram. PT = patellar tendinopathy; WOS = Web of Science.

A total of 1164 studies were screened for possible inclusion in this systematic review; 10 RCTs met our inclusion criteria and included in the review (Figure 1).

All the articles included in the present review involved 326 individuals, most of whom were athletes practicing various sports and aged between 18 and 55 years. Functionality with VISA-p was assessed in eight studies [8, 21, 23, 34–38], and pain was evaluated using a numeric scale in all studies except one [38]. The main features of these studies are summarized in Table 1. The quality assessment revealed that all the studies included were considered to be of high methodological quality (≥6 points about 10) (Appendix 2).

## 4. Review

A heterogeneity of results was found after performing the systematic review. On the one hand, MIT that do not infiltrate any substances such as DN or PNE showed a high increase of studies in the last years, although the studies published are still of low quality except for the one published by Dragoo et al. [21]. However, there was a protocol published by Lopez-Royo et al [41] analyzing the additional effects of both DN and PNE over EE whose results have not published yet, but that will increase the knowledge in this field. On the other hand, other therapies including the infiltration of substances, such as PRP therapy, were associated with a significant improvement in functionality and pain at posttreatment [21, 34–37] and at follow-up measurements [21, 35–37] and a significant decrease in hypoechogenicity and tear size at 6 months [37] in patients with PT, with no significant differences between one or two PRP injections.

In the systematic review, we found studies comparing different interventions, such as a study that compared PRP with ESWT, showing that the effectiveness of two injections of PRP was superior for pain and functionality measurements at posttreatment and at follow-up and for pain only at follow-up [35]. In other study, that compared PRP with DN therapy, controversial results were observed, as, in the short term (3 months), PRP obtained better results on pain and functionality compared to DN, whereas at 6 months, DN was more effective than PRP for functionality [21]. In this study, when comparing subjects at baseline, DN was more effective than PRP on levels of pain and function also at 6 months. Moreover, other RCTs have reported PRP as being less effective than other therapies [34, 37], like the study comparing PRP with tenocyte-like cell treatment at 6 months [37], with the latter showing improved effectiveness for functionality, pain, tendon thickness, hypoechogenicity, and tear size. In other recent study [34], comparing two different groups of leukocyte-poor PRP (LP-PRP) and leucocyte-rich PRP (LR-PRP) versus saline injection, 58% of the patients experienced an improvement of the VISA-p score at posttreatment regardless of their assigned treatment group. In this study, no significant differences were found between the three groups in the short term, failing to demonstrate any significant benefit of LP-PRP or LR-PRP over saline injection. Eventually, the effectiveness of the autologous blood (AB) injections was studied in a recent RCT [38], showing that both AB and saline groups experienced a significant improvement in symptoms, with nonsignificant statistical differences between groups.

Regarding pharmacological treatments, several studies [8, 23, 38–40] applied different drug infiltrations protocols, including steroids and sclerosing agents. A study comparing steroids with placebo showed an improvement at 6 months in the steroids group for pain measured with a numeric rating scale and a decrease of tendon thickness, maintaining this effect at 6 months [39]. Nevertheless, in a study comparing corticosteroid injections (CI) with EE and HSRT, all groups improved functionality, pain, and tendon thickness and swelling, with significant differences in favor of the CI group when compared with the other two exercise groups in the short term. However, these improvements were maintained at 6 months for both exercise groups but not for the group receiving corticosteroids [23]. Polidocanol was effective on functionality at 4 months posttreatment in one study [8]; however, no results were found in VAS measurements either in the short or long term [8, 40]. Furthermore, functional improvements were not maintained at long-term [8]. In a subsequent study of the same group with a 44-months follow-up, more than one-third of the group treated with polidocanol ended up in a surgical intervention because of the pain they suffered. Willberg et al. [40] in their study showed that the group receiving arthroscopic treatment found better improvements in pain and satisfaction compared to baseline as opposed to the cited polidocanol group at 6 weeks [40]; however, once again, the results were lost in the follow-up period (2, 6, and 12 months).

### 4.1. Meta-Analysis

The only MIT with enough RCTs to carry out a meta-analysis was PRP. For this reason, we included all articles with at least one group consisting of PRP injection treatment. Finally, five RCTs were included in the meta-analysis (Figure 1).

For the meta-analysis, different cutoff points were established. Regarding the dose of treatment received, three RCTs [21, 36, 37] applied doses equal to or greater than 4 mL of PRP, and two RCTs [34,35] employed three different injections, applying doses less than 4 mL. These same subgroups were obtained when comparing the duration of EE performed by the subjects of the PRP group. When the dose was bigger than 4 mL of PRP, a program of over 6 weeks of exercise was continued; however, when the dose was less than 4 mL, the exercise program lasted 6 weeks or less. Regarding the mean baseline score of the VISA-p, the following subgroups were defined: subjects with less than 48 points [21, 34] and subjects with more than 48 points [34–37].

### 4.2. Intergroup Differences

**Figure 2:**
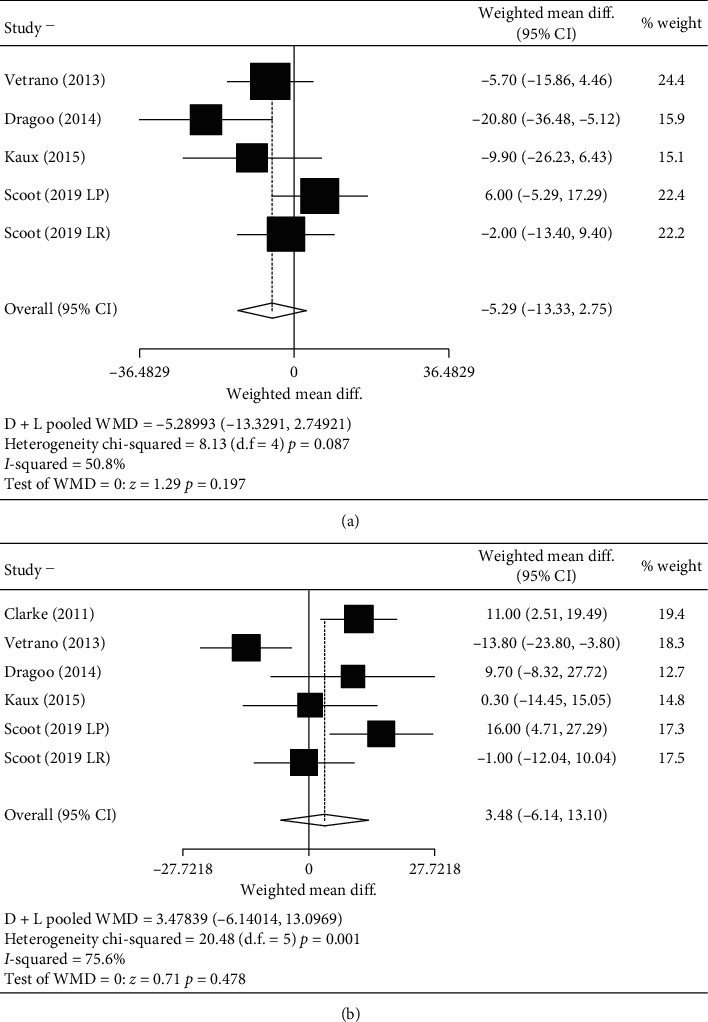
Analysis VISA-p mean differences. (a) Analysis VISA-p prepost mean difference. (b) Analysis of VISA-p pre-follow-up mean difference.

**Figure 3:**
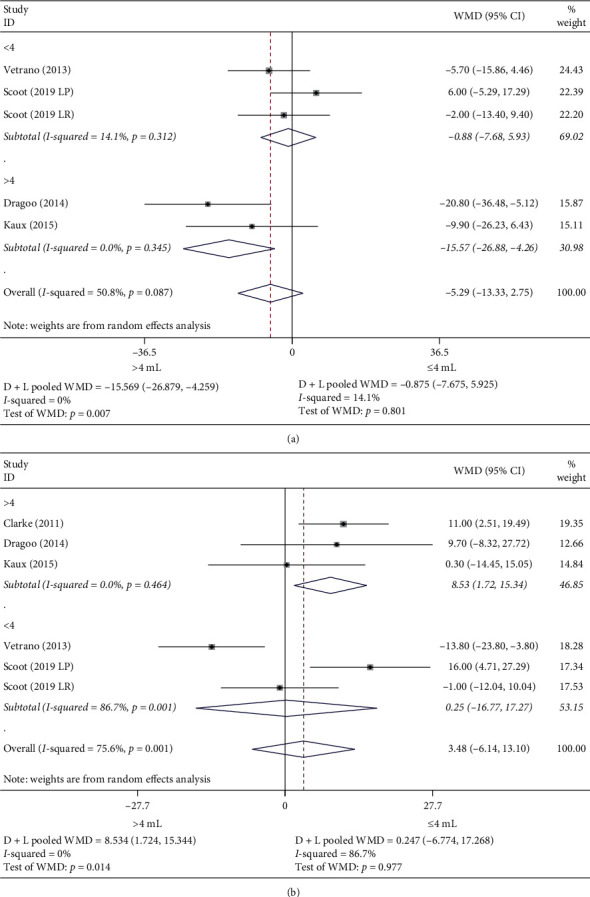
Subgroup analysis VISA-p mean difference. (a) Subgroup analysis VISA-p prepost mean difference. (b) Subgroup analysis VISA-p pre-follow-up mean difference.

There was no difference between groups in the case of the VISA-p after treatment or at follow-up (Figures 2(a) and 2(b)). However, in those studies where the dose was greater than 4 mL and the duration of the exercise program was longer than 6 weeks, treatment with PRP was better than other therapies after treatment. Surprisingly, the opposite happened in the follow-up (Figures 3(a) and 3(b)).

**Figure 4:**
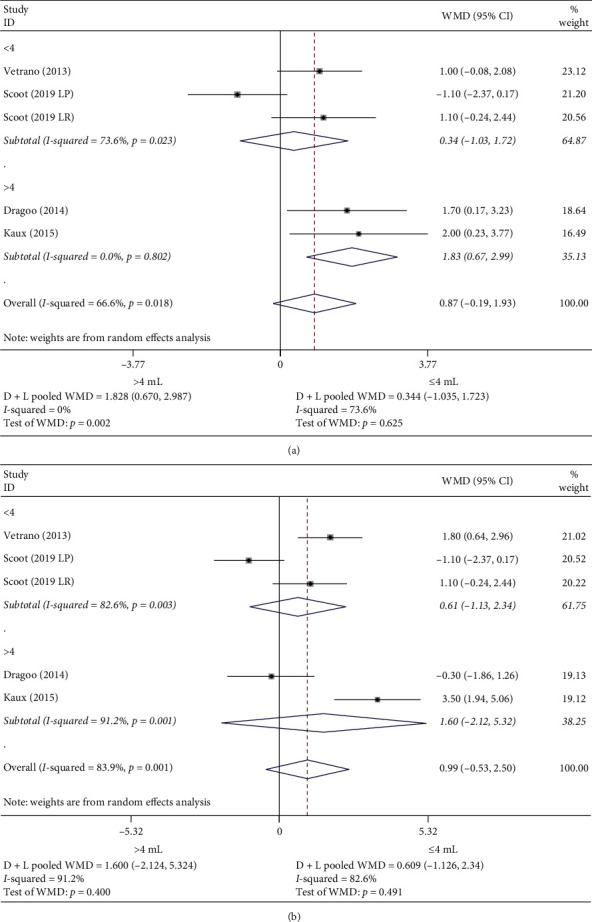
Subgroup analysis VAS mean difference. (a) Subgroup analysis VAS prepost mean difference. (b) Subgroup analysis VAS pre-follow-up mean difference.

Concerning the effectiveness of treatment in the management of pain measured by VAS, PRP was superior to other treatments in the case of doses greater than 4 mL and exercise programs of more than 6 weeks duration after treatment. In the follow-up, there were no differences between groups in any of the situations (Figures 4(a) and 4(b)).

### 4.3. Intragroup Differences for the PRP Group

**Figure 5:**
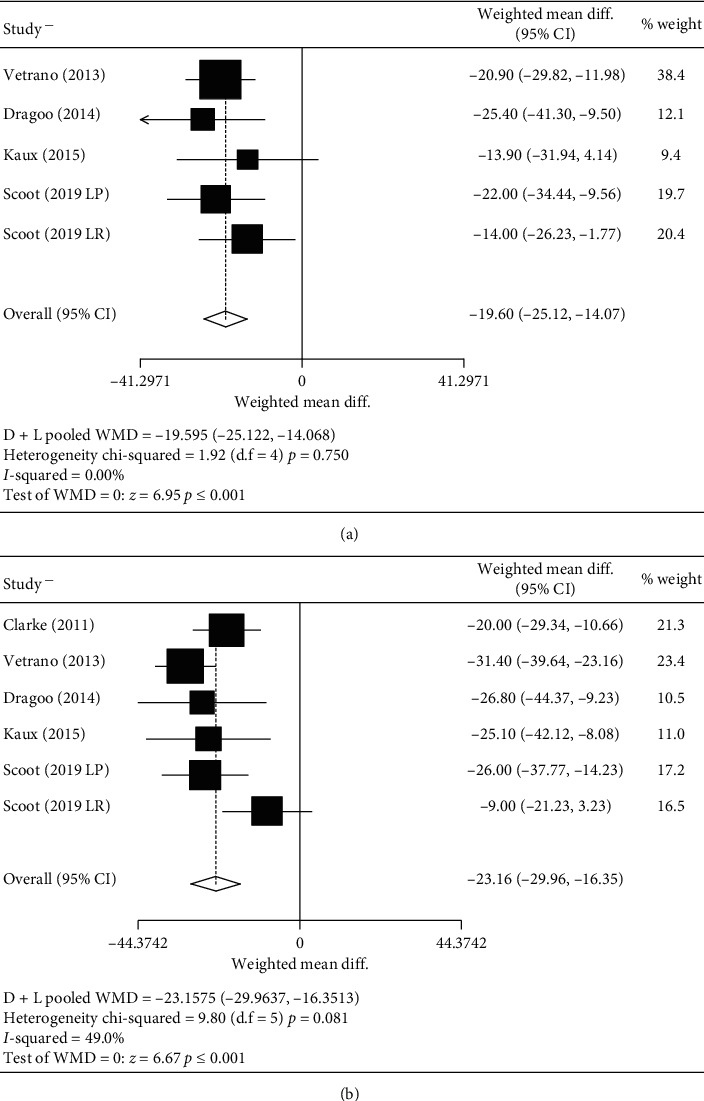
Intragroup VISA-p mean difference in the PRP group. (a) Intragroup prepost VISA-p mean difference in the PRP group. (b) Intragroup pre-follow-up VISA-p mean difference in the PRP group.

**Figure 6:**
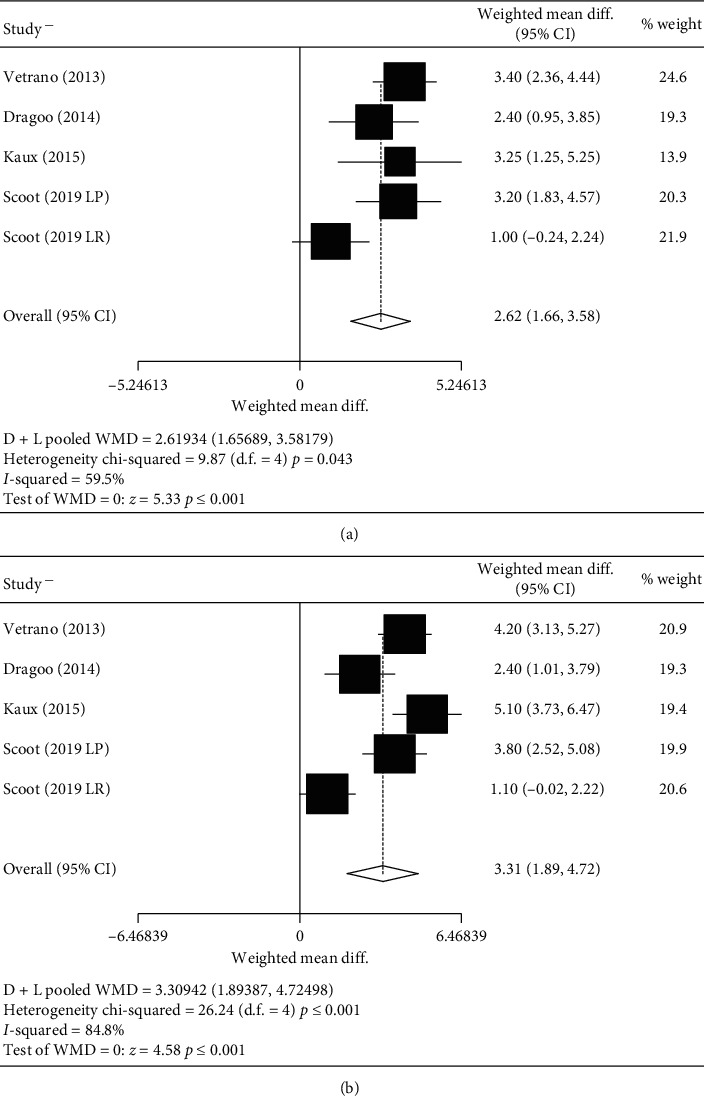
IntragroupVAS mean difference in the PRP group. (a) Intragroup prepost VAS mean difference in the PRP group. (b) Intragroup pre-follow-up VAS mean difference in the PRP group.

Concerning the intragroup analysis of those patients treated with PRP, treatment was effective both after treatment and during the follow-up, according to both measurement scales: the VISA-p (Figures 5(a) and 5(b)) and VAS (Figures 6(a) and 6(b)). In the case of pain, heterogeneous findings were found; however, the results of all studies suggest an improvement after treatment.

## 5. Discussion

The main finding of this study was that all studies analyzing MIT, such as PRP, DN, or cells, when combined with exercise, were found to be effective for PT at posttreatment and follow-up. This systematic review has identified ten RCTs assessing the effectiveness of MIT in the treatment of PT. A meta-analysis was conducted with five RCTs that included PRP injection. It was not possible to perform meta-analysis with other MIT due to a lack of data for conducting statistical analysis. All studies selected in this review had a high-quality level.

MIT can be divided into two different groups, depending on whether they inject any substances or not. For the injection of substances, a differentiation can be made between infiltrating pharmacological agents or nonpharmacological ones. In our review, all injection therapies with pharmacological agents (i.e., steroids, corticosteroids, and polidocanol) obtained good clinical effects in the short term, even for the decrease of the tendon diameter [8, 23, 40]; however, in the long term, results were poor [8]. These findings are in line with the conclusions of previous systematic reviews for the treatment of other tendon disorders affecting other areas of the body [42,43]. Furthermore, the role of steroids in the management of tendinopathy is also controversial, suggesting the relapse of symptoms in the long term [43]. Also, steroid injections may produce undesirable side effects, such as tendon rupture, as reported elsewhere [42, 44].

Concerning infiltration with nonpharmacological agents such as PRP, in this meta-analysis, as reported by several RCTs [25, 26, 42, 45], PRP was found to be an effective technique for PT treatment, and these effects were maintained in the long term. Nonetheless, these studies conclude the need to perform further quality RCTs including placebo treatments to elucidate their true effects. Moreover, this study has found that other MIT such as tenocyte-like cells or DN may have better results in the long term for this pathology. Studies focusing on other tendinopathies, which compare PRP versus MIT that do not infiltrate substances, are available. In the case of the rotator cuff tendinopathy, PRP resulted in better results at 3 and 6 months when compared with DN [46]. Nonetheless, a single study comparing PRP with DN in PT [21] showed better short-term results in the case of PRP but the opposite at 6 months follow up. These differences may be due to the comparison of different tendons and a different methodology, as the aforementioned studies differ in the number of injections (1–8) and dosage (6 mL versus 3 mL of PRP and 10 versus 40–50 needle insertions for DN group) in the PT and rotator cuff tendinopathy, respectively.

In line with the study by Dragoo et al. [21] and Clarke et al. [37], in our meta-analysis according to the subgroup analysis, we can affirm that in doses of less than 4 mL of PRP, there is no difference between groups, and when the dose is more than 4 mL of PRP, in the long term, other treatments are more effective than PRP. One of such treatments is skin-derived tenocyte-like cells, which has proven to be effective for the recovery of other tendinopathies, such as lateral epicondylitis [47]. Therefore, this appears to be a good option for the treatment of tendinopathies, although further studies are required to demonstrate the effectiveness of this same. According to some studies [16, 36, 45], it seems that different PRP preparations, timing, and frequency of injections should be considered in future studies as these factors could, at least in part, explain the differences in the effectiveness of this technique. A recent meta-analysis compared the application of one or two PRP injections, showing that two injections do not show better results than a single injection [16]. In our meta-analysis, we have gone further and have tried to clarify not only the number of infiltrations but also what is the dose producing the vest results. Thus, our study provides novel and valuable information regarding the PRP technique and what appears to be the best dose.

In the most recent RCT (2019) [34], aimed at examining the differences between the concentration of leukocytes injected in the PRP, a platelet-rich sample was compared with a platelet-poor sample and a final control group with a saline solution. This study revealed that there are no differences between the groups neither in the short nor in the long term. Along these lines, Resteghini et al. [38] compared the effectiveness of AB versus saline and found no differences between groups. Finally, this review is in line with a previous study [21] that reported that PRP was not superior to placebo or DN at a 6-month follow-up for tendinopathy treatment. These results may support the hypothesis by Vetrano [35] who proposed that needling on tissues with tendinopathy may provide cellular and humoral mediators, which promote the healing of tissues.

Although there are no RCT analyzing the effectiveness of MIT like PNE, there has been an increasing number of publications achieving excellent results in the last years [4, 18–20]. However, it is necessary to develop RCTs analyzing not only the effect but also comparing the effectiveness between different MIT such as DN and PNE. Fortunately, this review found two high quality protocols published that aim to compare the effects of PNE and DN in PT [41] and plantar heel pain [48], although results are not available yet.

The studies previously mentioned in this review testing cell therapy, PRP, DN, and AB [21, 31, 34, 35, 37, 38] were accompanied by a standardized program of exercise during treatment, most of them with EE which is until now, the gold standard technique for the treatment of this injury. However, several studies have shown that HSR is more effective than EE [23]. Further studies are necessary to redefine the gold standard of conservative treatment of this pathology. In the subgroup analysis of functionality and pain in PRP treatment, RCTs which carried out EE programs over 6 weeks showed greater improvements than those which had a duration of less than 6 weeks. Further research is needed to elucidate the ideal type and duration of exercise programs for the different subtypes of patients with different levels of functionality.

The current systematic review and meta-analysis had several limitations due to the inherent biases of the included studies. Thus, caution should be taken when interpreting the findings. First, there is a scarcity of RCTs concerning certain MIT that do not rely on the infiltration of substances (i.e., DN or PNE). This hinders the possibility of performing a meta-analysis with all the MIT. Second, a standardization of treatment may be necessary (number of doses, frequency, and PRP amount). Third, publication bias may be present, as only English or Spanish language studies were included, and only 5 databases were searched; however, in order to decrease publication bias, a monthly update of the published papers on databases were performance.

## 6. Conclusions

In conclusion, the most important finding of the present study was that all studies analyzing MIT, such as PRP, DN, or cells, when combined with exercise, were found to be effective for PT at posttreatment and follow-up. Moreover, the PRP technique with doses greater than 4 mL together and combined with an exercise program lasting over 6 weeks obtained better results in functionality and pain compared to other treatments in the short-term. However, in the long-term, DN and skin-derived tenocyte-like cells are more effective than PRP. In addition, although the infiltration of drugs was effective at posttreatment, these improvements were not maintained over time and could have secondary effects.

## Figures and Tables

**Table 1:** Study characteristics of the included trials.

Study (y)	Participants (*N*, age, sex)	Intervention	Outcomes	Results	Conclusion
Dragoo et al. (2014) [21]	DN (*n* = 12, 40 (SD 14) yrs, 100% M)	A nurse obtained 55 mL of peripheral blood that was discarded.	Functionality: VISA-p, Tegner/Lysholm. Pain: VAS. Others: SF-12. Temporality: pre/post (12 wks)/follow-up (26 wks).	**DN**: significant improvement from baseline on Lysholm at 12 wks and on VISA-p, Tegner, Lysholm, and VAS at 26 wks. **PRP**: significant improvement from baseline on VISA-p, Lysholm, and VAS at 12 wks and on VISA-p and VAS at 26 wks. **DN vs. PRP**: significant improvement in PRP vs. DN on VISA-p at 12 wks and DN vs. PRP on Lysholm at 26 wks.	The PRP group showed a significant improvement compared with the DN group at 12 wks, but the difference between groups was not significant at 26 wks. Lysholm scores were not significantly different between groups at 12 wks, but the DN group had improved significantly more than the PRP group at 26 wks.
		3 mL of 0.25% bupivacaine with 1 : 100,000 epinephrine were then injected subcutaneously using a sterile technique to anesthetize. Subsequently, 10 mechanical needle insertions were performed in the area of the tendinopathy. Standardized a 5-phase program of EE during 12 wks.			
	PRP (*n* = 9, 28 (SD 8) yrs, 89% M)	A nurse obtained 55 mL of peripheral blood, and it was processed. Then, 3 mL of 0.25% bupivacaine with 1 : 100,000 epinephrine was subcutaneously injected using a sterile technique to anesthetize. This was followed by 6 mL infiltration of leukocyte-rich PRP. Finally, 10 mechanical needle insertions were performed in the area of the tendinopathy. Standardized a 5-phase program of EE during 12 wks.			

Clarke et al. (2010) [37]	Cells (*n* = 33); total sample: 36 yrs (20–51)	Skin-biopsy: 4-mm skin sample. 1 infiltration of isolated and amplified cells + plasma. Standardized program of increased eccentric loading and stretching exercises during 6 mo.	Functionality: VISA-p. Others: US (tendon thickness, hypoechogenicity, intrasubstance tears, and neovascularity). Temporality: pre/post (6 wks)/follow-up (3 mo and 6 mo).	**Cells**: significant improvement from baseline on VISA-p, tendon thickness, hypoechogenicity and tear size at 6 mo. **PRP**: significant improvement from baseline on VISA-p, hypoechogenicity, and tear size at 6 mo. **Cells vs. PRP**: significant improvement in cells vs. PRP on VISA-p at 6 mo.	Ultrasound-guided injection of autologous skin-derived tendon-like cells can be safely used at 6 mo to treat PT, with a faster treatment response and significantly greater improvements in pain and function than with plasma alone.
	PRP (*n* = 27); total sample: 36 yrs (20–51)	Skin biopsy: 4-mm skin sample. 1 infiltration of centrifuged autologous whole blood (8 mL). Standardized program of increased eccentric loading and stretching exercises during 6 mo.			

Kongsgaard M. et al. (2009) [23]	CORT (*n* = 13, 34.3 (SD 10.0) yrs)	Injections of 1 mL of 40 mg/mL methylprednisolone in 0.5 mL lidocaine (1%). Second injection was 4 wks later.	Functionality: VISA-p. Pain: VAS. Others: US (tendon swelling) and CD (vascularization). Temporality: pre/post (12 wks) /follow-up (6 mo).	**CORT**: significant improvement from baseline on VISA-p, VAS, tendon thickness, and color area at 12 wks. **ECC**: significant improvement on VISA-p and VAS at 12 wks and 6 mo. **HSRT**: significant improvement on VISA-p, VAS, tendon thickness, and color area at 12 wks and on VISA-p and VAS at 6 mo. **HSRT vs. EE vs. CORT**: VISA-p improvement from baseline to 6 mo was significantly higher in HSRT and EE than CORT. VAS improvement from baseline to 6 mo was significantly greater in HSRT compared with CORT.	CORT has good clinical effects at 3 mo but poor results at 6 mo in PT. HSRT and EE have good clinical effects at 3 and 6 mo accompanied by an improvement in the pathology.
	EE (*n* = 13, 31.3 (SD 8.3) yrs)	3 x 15 repetitions of eccentric unilateral squats on a 25° decline board twice daily for 12 wks.			
	HSRT (*n* = 13, 31.7 (SD 8.5) yrs)	15 repetitions maximum of 3 bilateral exercises: squat, leg-press, and hack squat three times a week for 12 wks. RM wks 1, 12 RM wks 2-3, 10 RM wks 4-5, 8 RM wks 6–8, and 6 RM wks 9–12.			

Hoksrud et al. (2006) [8]	TG (*n* = 17, 25.4 (SD 7.5) yrs)	First treatment: polidocanol 10 mg/mL 3 times maximum in 4 mo. Second treatment (at 4 mo): maximum 3 sclerosing polidocanol injections conditioned to patient willingness.	Functionality: VISA-p. Pain: VAS during squat testing. Satisfaction: 100-mm long scale. Temporality: pre/post (4 mo)/follow-up (8 and 12 mo).	**TG**: significant improvement on VISA-p at 4 mo. **CG**: significant improvement on VISA-p at 8 and 12 mo. **TG vs. CG**: more satisfaction in TG compared with the CG at 4 mo.	Sclerosing injections with polidocanol resulted in a significant improvement in knee function and reduced pain measured with VISA-p in patients with PT.
	CG (*n* = 16, 24.3 (SD 4.5) yrs)	First treatment: lidocaine with adrenaline (xylocaine-adrenalin) (5 mg/mL + 5 g/mL). Second treatment (at 4 mo): maximum 3 sclerosing polidocanol injections conditioned to the patients' willingness.			

Fredberg et al. (2004) [39]	Steroids (*n* = 12); total sample: 28.4 yrs (18–47)	3 infiltrations (0, 7, and 21 days). Steroid injection (contained 3.5 mL of 10 mg/mL lidocaine and 0.5 mL Kenalog containing 20 mg triamchinolone in a 5-mL syringe).	Pain: NRS, algometry. Others: US (tendon thickness). Temporality: pre (7 days)/post (21 days)/follow-up (28 days and 6 mo).	**Steroids**: significant improvement from baseline on tendon diameter at 7 days, 21 days, and 6 mo and on pressure-pain at 21 days. **Steroids vs. Placebo**: significant improvement in steroids vs. placebo on NRS at 6 mo.	Ultrasonographically guided injection of long-acting steroid can normalize the ultrasonographic pathological lesions in the PT.
	Placebo (*n* = 12); total sample: 28.4 yrs (18–47)	3 infiltrations (0, 7, and 21 days). Placebo injection contained 3.5 mL of 1% lidocaine and 0.5 mL of 20% intralipid in a 5-mL syringe			

Vetrano et al. (2013) [35]	PRP (*n* = 23, 26.9 (SD 9.1) yrs, 86.9% M)	10 mL of venous blood was collected. 2 autologous PRP injections (2 mL) over 2 wks. Standardized stretching and muscle strengthening protocol (2 wks).	Functionality: VISA-p. Pain: VAS. Temporality: pre/follow-up (2, 6, and 12 mo).	**PRP**: significant improvement from baseline on VISA-p and on VAS at 2, 6, and 12 mo. **ESWT:** significant improvement from baseline on VISA-p and on VAS at 2, 6, and 12 mo. **PRP vs. ESWT:** significant improvement in PRP vs. ESWT in VISA-p and VAS at 6 and 12 mo.	Therapeutic injections of PRP lead to better clinical results at 6–12 mo compared with focused ESWT in the treatment of PT in athletes.
	ESWT (*n* = 23, 26.8 (SD 8.5) yrs, 73.9% M)	3 sessions in 48–72 h intervals. 2.400 impulses with an energy flux density of 0.17–0.25 mJ/mm^2^. Standardized stretching and muscle strengthening protocol (2 wks).			

Kaux et al. (2015) [36]	PRP (*n* = 10, 31.1 (SD 10, 4) yrs)	1 PRP injection (6 mL) + standardized EE 15 x 3 times/5 times a week.	Functionality: VISA-p. Pain: VAS and algometer. Others: isokinetic (Cyber Norm), IKDC and US. Temporality: pre/6 wks/3 mo.	**1 PRP:** significant improvement from baseline in VAS, pressure algometer, IKDC, and VISA-p at 3 mo. **2 PRP:** significant improvement from baseline in VAS, pressure algometer, IKDC, and VISA-p at 3 mo. **1 PRP vs. 2 PRP:** significant improvement in 1 PRP vs. 2 PRP in pressure algometer and IKDC at 3 mo. And an increase of the sagittal hypoechoic area.	A local infiltration of PRP associated with EE is efficient to improve symptoms of PT. The application of 1 or 2 infiltration of PRP did not reveal any difference between groups.
	PRP (*n* = 10 29.5 (SD 5, 87) yrs)	2 PRP injections (6 mL) 1 one between them + standardized EE 15 x 3 times/5 times a week.			

Willberg et al. (2011) [40]	Group 1 (*n* = 26, 27.0 (SD 7.6) yrs)	Sclerosing polidocanol injections (2 mL), maximum 3 times (at least 6 weeks in between).	Pain: VAS at rest and during sport activity. Satisfaction: 100-mm long scale. Temporality: pre/post (6 wks)/follow-up (2, 6, and 12 mo).	**Group 2:** significant improvement from baseline in VAS at rest and with activity and in satisfaction posttreatment.	Patients treated with arthroscopic shaving had less pain and were more satisfied with the treatment result.
	Group 2 (*n* = 26, 26.6 (SD 7.6) yrs)	Arthroscopic shaving was performed under local anesthesia. Standard anteromedial and anterolateral portals.			

Resteghini et al. (2016) [38]	AB (*n* = 11, 42 yrs)	2 mL of AB was extracted. 2 mL AB injection + EE 3 mo	Functionality: VISA-p. Pain: SF-MPQ and VA. Temporality: pre/post (6 wks)/follow-up (3, 6, and 12 mo)	AB: significant improvement VAS, VISA-p, SF-MPQ at 1 mo/3 mo/1 yr. Saline group: significant improvement VAS, VISA-p, and SF-MPQ at 1 mo/3 mo/1 yr. AB vs. saline: no significant differences.	AB and saline groups experienced a significant improvement in symptoms. However, when the results were compared, there was no statistical difference between the 2 groups.
	Saline (*n* = 11, 39 yrs)	2 mL of AB was extracted. 2 mL saline injection + EE 3 mo			

Scott et al. (2019) [34]	LP-PRP (*n* = 20, 33 (SD 7.3) yrs)	52 mL of venous blood was collected. 2% hematocrit in 3.5 mL PRP + HSRT 3 times/wk during 6 wks.	Functionality: VISA-p. Pain: NRS. Temporality: pre/post (6 wks)/follow-up (3, 6, and 12 mo)	No significant difference between groups in any time.	Combined with an exercise-based rehabilitation program, a single injection of LR-PRP or LP-PRP was no more effective than saline for the improvement of PT symptoms.
	LR-PRP (*n* = 20, 32 (SD 9.8)	52 mL of venous blood was collected. 15% haematocrit in 3.5 mL PRP + HSRT 3 times/wk during 6 wks.			
	Saline (*n* = 21, 31 (SD 7.9)	3.5 mL saline + HSRT 3 times/wk during 6 wks.			

AB, autologous blood; Cells, prepared in laboratory (cells of collagen and plasma); CD, Color Doppler; CG, control group; CORT, peritendinous corticosteroid injections; DN, dry needling; EE, eccentric decline squat training; ESWT, extracorporeal shock wave therapy; HSRT, heavy slow resistance training; IKDC, international knee documentation committee form; LP-PRP, leukocyte poor PRP; LR-PRP, leukocyte rich PRP; M, masculine; mo, months; MRI, magnetic resonance imaging; *N*, number; NRS, numeric rating scale; PRP, platelet-rich plasma; PT, patellar tendinopathy; RCT, randomized control trial; SF-12, Short Form–12; TG, treatment group; US, ultrasound; VAS, visual analog scale; VISA-p, Victorian Institute of Sport Assessment of Patellar; wks, weeks; yr, years.

## Data Availability

The data used to support the findings of this study are included within the supplementary information file.

## Abbreviations

AB: Autologous blood
CD: Color Doppler sonography
CI: Corticosteroid injections
DN: Dry needling
EE: Eccentric exercise
ESWT: Extracorporeal shockwave therapy
HSR: Heavy slow resistance exercises
LP-PRP: Leukocyte-poor PRP
LR-PRP: Leucocyte-rich PRP
MIT: Minimally invasive techniques
MRI: Magnetic resonance imaging
PNE: Percutaneous needle electrolysis
PRISMA: Preferred Reporting Items for Systematic Review and Meta-Analysis
PRP: Platelet-rich plasma
PT: Patellar tendinopathy
RCT: Randomized controlled trial
SD: Standard deviation
VAS: Visual analog scale
VISA-p: Victorian Institute of Sport Assessment of Patellar Questionnaire
WOS: Web of Science.

## Supplementary Materials

Supplementary Materials Appendix 1: search strategy in databases. Appendix 2: methodological quality scores using the PEDro scale.
