# Supplementary material for: The Effectiveness of Minimally Invasive Techniques in the Treatment of Patellar Tendinopathy: A Systematic Review and Meta-Analysis of Randomized Controlled Trials
Source: Evid Based Complement Alternat Med. 2020 Sep 5;2020:8706283. doi: 10.1155/2020/8706283 (PMC7492866; doi:10.1155/2020/8706283)
Supplement: Supplementary Materials — Appendix 1: search strategy in databases. Appendix 2: methodological quality scores using the PEDro scale. [file 8706283.f1.zip › mat.8706283.v1 (2).docx]

**Appendix 1. Search strategy.**

(patellar ten* OR patellar ligament OR jumper’s knee OR chronic patellar ten*) AND (dry need* OR intratissue percutaneous electrolysis OR acupuncture OR electroacupuncture OR mesotherapy OR injection OR injectabl* OR puncture OR infiltrat*) without limited years.
