# Supplementary material for: The Effectiveness of Minimally Invasive Techniques in the Treatment of Patellar Tendinopathy: A Systematic Review and Meta-Analysis of Randomized Controlled Trials
Source: Evid Based Complement Alternat Med. 2020 Sep 5;2020:8706283. doi: 10.1155/2020/8706283 (PMC7492866; doi:10.1155/2020/8706283)
Supplement: Supplementary Materials — Appendix 1: search strategy in databases. Appendix 2: methodological quality scores using the PEDro scale. [file 8706283.f1.zip › Appendix 2. Methodological quality scores using PEDro scale.docx]

**Appendix 2. Methodological quality scores using PEDro scale.**

| REFERENCES | Items in the PEDro Scale | | | | | | | | | | | |
| --- | --- | --- | --- | --- | --- | --- | --- | --- | --- | --- | --- | --- |
|  | **1** | **2** | **3** | **4** | **5** | **6** | **7** | **8** | **9** | **10** | **11** | **TOTAL** |
| Dragoo JL et al. 2014 [22] | 1 | 1 | 1 | 1 | 1 | 1 | 1 | 0 | 1 | 1 | 1 | 10 |
| Clarke AW et al. 2011 [38] | 1 | 1 | 1 | 0 | 1 | 1 | 1 | 1 | 1 | 1 | 1 | 9 |
| Kongsgaard M et al. 2009 [24] | 1 | 1 | 1 | 1 | 1 | 0 | 0 | 0 | 1 | 1 | 1 | 7 |
| Hoksrud A et al. 2011 [8] | 1 | 1 | 1 | 1 | 1 | 1 | 1 | 1 | 1 | 1 | 1 | 10 |
| Fredberg U et al. 2004 [40] | 1 | 1 | 1 | 1 | 1 | 1 | 1 | 1 | 1 | 1 | 1 | 10 |
| Vetrano M et al. 2013 [36] | 1 | 1 | 1 | 1 | 0 | 0 | 1 | 1 | 1 | 1 | 1 | 8 |
| Kaux et al. 2016 [37] | 1 | 1 | 1 | 1 | 0 | 0 | 0 | 1 | 1 | 1 | 1 | 8 |
| Willberg L et al. 2011 [41] | 1 | 1 | 1 | 1 | 0 | 0 | 1 | 1 | 1 | 1 | 1 | 8 |
| Resteghini 2016 [39] | 1 | 1 | 1 | 1 | 1 | 1 | 1 | 1 | 1 | 1 | 1 | 10 |
| Scott 2019 [35] | 1 | 1 | 1 | 1 | 1 | 0 | 1 | 1 | 1 | 1 | 1 | 9 |

1=YES 0= NO
